# Binary Mixtures of Selected Bisphenols in the Environment: Their Toxicity in Relationship to Individual Constituents

**DOI:** 10.3390/molecules23123226

**Published:** 2018-12-06

**Authors:** Katarzyna Owczarek, Błażej Kudłak, Vasil Simeonov, Zofia Mazerska, Jacek Namieśnik

**Affiliations:** 1 Department of Analytical Chemistry, Faculty of Chemistry, Gdańsk University of Technology, 11/12 Narutowicza Str., 80-233 Gdańsk, Poland; katarzyna.owczarek.90@gmail.com (K.O.); jacek.namiesnik@pg.edu.pl (J.N.); 2 Department of Analytical Chemistry, Faculty of Chemistry and Pharmacy, University of Sofia, St. Kliment Ohridski, 1164 Sofia, 1 James Bourchier Blvd., Bulgaria; vsimeonov@chem.uni-sofia.bg; 3 Department of Pharmaceutical Technology and Biochemistry, Faculty of Chemistry, Gdańsk University of Technology, 11/12 Narutowicza Str., 80-233 Gdańsk, Poland; zofia.mazerska@pg.edu.pl

**Keywords:** bisphenol A analogues, Microtox^®^, XenoScreen YES/YAS, model deviation ratio

## Abstract

Bisphenol A (BPA) is one of the most popular and commonly used plasticizer in the industry. Over the past decade, new chemicals that belong to the bisphenol group have increasingly been used in industrial applications as alternatives to BPA. Nevertheless, information on the combined effects of bisphenol (BP) analogues is insufficient. Therefore, our current study aimed to find the biological response modulations induced by the binary mixtures of BP compounds. We determined the toxicity levels in Microtox and XenoScreen YES/YAS assays for several BP analogs alone, and for their binary mixtures. The results obtained constituted the database for chemometric intelligent data analysis to evaluate the possible interactions occurring in the mixtures. Several chemometric/biophysical models have been used (concentration addition—CA, independent action—IA and polynomial regression calculations) to realize this aim. The best fitting was found for the IA model and even in this description strong evidence for synergistic behaviors (modes of action) of some bisphenol analogue mixtures was demonstrated. Bisphenols A, S, F and FL were proven to be of significant endocrine threat (with respect to XenoScreen YES/YAS assay); thus, their presence in mixtures (including presence in tissues of living organisms) should be most strictly monitored and reported.

## 1. Introduction

Over the past decade, new chemicals that belong to the bisphenol group have increasingly been used in industrial applications as an alternative to bisphenol A, but current knowledge of their environmental and biological impact is still limited. Initially, the occurrence of bisphenol analogues was poorly researched, but in recent years, this chemical group has been attracting more scientific attention. The number of studies proving that bisphenols are present in different elements of the environment is growing, but to the best of our knowledge, there is no study focused on the assessment of the possible interactions occurring between these chemicals.

The group of bisphenols consists of chemicals that contain two p-hydroxyphenyl functionalities in their molecular structure. The most widely known analogue—BPA—Is a synthetic chemical used for a broad spectrum of commercial applications worldwide. BPA was synthesized for the first time in 1891, and it has been used in industries for the commercial production of epoxy resins since the early 1950s. In the polymerization process, BPA is used to create polycarbonate plastic—A very durable and hard material [1]. BPA has reached an annual global production of six million metric tons and this is predicted to increase. Epoxy resins and polycarbonates are used in the manufacturing of a large number of everyday items; the human exposure routes to BPA include dermal, oral and inhalation intake.

BPA has been comprehensively studied for its impact on human health and is well known for its estrogenic activity. Furthermore, a vast number of other adverse effects have been proven, including neural and developmental disorders [2,3], alternation of thyroid function [4], metabolic disorders [5], and suspicion of increasing the risk of Parkinson disease [6]. Moreover, bisphenol A is also a hazard to the environment, especially to aquatic ecosystems, due to its ubiquitous presence. Because of ecological and health concerns regarding BPA, new BPA-related chemicals were considered to be safer alternatives to partially replace BPA in industrial applications. A total of 16 bisphenols have been documented to be industrially applied [7,8,9].

Bisphenol S and bisphenol F are currently the most commonly used BPA substitutes, predominantly in the manufacturing of epoxy resins, polyesters and polycarbonate plastics. Other bisphenol analogues are also used in the plastic industry to produce a broad spectrum of products, such as dental sealants, pesticides, thermal papers, food containers’ inner coatings, toys lacquers, and powder paints. Supplementary Table S1 presents basic information about tested bisphenols, their chemical structures, IUPAC names, and most common applications.

The first regulatory standard for BPA was established by the Environmental Protection Agency (EPA) in 1988, and the oral reference admissible dose was assessed to be 50 mg/kg/day [10]. Because of growing concerns about health and developmental issues, and continually increasing occurrence in the environment, in 2010, baby bottles containing BPA were prohibited by the Canadian government; the European Union followed suit in 2011 [11]. Since BPA is permitted to be used in food contact materials (under the regulation framework 10/2011/EU), in 2015, the tolerable daily intake (TDI) of BPA was reduced from 50 mg per kg to 4 mg per kg of body mass.

These regulations on BPA use and production created the necessity to develop and produce safer alternatives [12,13] that can serve as plasticizers, especially in the case of BPA-free products. BPS and BPF are the most broadly used analogues, but recently there is a growing amount of scientific data, indicating that humans may be exposed to other analogues such as BPE, BPBP, BPM, BPP, BPZ, BPAF [14,15,16]. Environmental issues are emerging as well, since a vast number of bisphenol analogues has been found in different elements worldwide. BPA, BPS, BPF, BPAF, TCBPA, BPAP, BPFL, and BPZ were detected in the environmental samples of water bodies and sediments [17,18]; BPA, BPE, BPF, BPS, BPP, BPZ TBBPA, TCBPA, and BPAF were detected in sludge and indoor dust [7,19,20,21]; and BPA, BPB, BPE, BPF, BPP, BPS, BPZ BPAF, and BPAP were detected in foodstuff [21,22]. Even though other bisphenol analogues show many similarities to BPA, these chemicals do not fall under any legislative regulations. It is important to highlight that in the past decade, scientific knowledge about the modes of action of many of these chemicals has expanded significantly. Despite this fact, relatively old safety standards, based on a threshold-dose model, are still valid. There is also strong evidence indicating that other chemicals that belong to the bisphenol group exert similar or even stronger endocrine and toxic effects than BPA, but their use in manufacturing everyday use products is still not regulated.

In most cases, modern analytical chemistry plays a key role in identifying and quantifying bisphenols in different matrices. High performance liquid chromatography or gas chromatography coupled with tandem mass spectrometry are commonly used for this purpose [15,16,21]. On the other hand, when the main goal is to assess the biological risk, bioassays are the best tools to distinguish the types of interactions and the modes of action of chemicals towards living organisms. Available scientific data indicate various toxic effects of bisphenol analogues. These data include endocrine effects, genotoxic action, neurotoxicity, and reproductive disruption [7]. Most of the BPs exhibit estrogenic potential similar to or even stronger than that of bisphenol A (except from BPF, BPS and BPC) [23]. An antiandrogenic mode of action has also been observed for BPA, BPF, BPE and BPB [7]. Most bisphenols may also have an impact on gene expression processes, especially those associated with enzymatic proteins, which influence fetal development. BPA was also proven to affect genes related to the immune system [24].

As confirmed by numerous studies, bioassays are the most efficient effect-based tools to learn about the combined biological effects of complex mixtures. They have the potential to elucidate the relationship between chemical contamination and ecological status of a given object of interest [25]. They can also be easily used as a regulatory reference for deriving environmental quality standards under the Water Framework Directive [26].

The model deviation ratio approach is a reliable tool with which to determine the possible synergistic or antagonistic effects of environmental stressors studied. To assess the toxicity of bisphenol A analogues (when present in binary mixture with BPs), the MDR approach was applied to the experimental results of ecotoxicity studies with Microtox^®^; subsequently, a correlation with XenoScreen YES/YAS was performed [27,28,29].

Considering the referenced information given before, the problem of BPs co-occurrence in the environment is clearly visible. Unfortunately, the numerous implications of their presence in complex mixtures with other environmental stressors are not understood yet. Bearing in mind all above, the goal of this study was to evaluate the toxicity of the series of BPs (alone and in their binary mixtures) with the *Vibrio fischeri* bacteria (as a non-target environmental prokaryote model) and gather data regarding plausible endocrine potentials. Simple analysis of the structure-activity led to preliminary conclusions on the role of bisphenol structure elements in their toxicity. Furthermore, the reliable modeling techniques that were used in this study allowed us to determine the possible interactions (antagonistic or synergistic) between the analytes that may occur in the real environmental samples and that have an influence on their final biological potency. To the best of our knowledge, the approach to evaluate the environmental toxicity of the single analytes and binary mixtures of the broad spectrum of the chemicals belonging to the bisphenols group was undertaken for the first time in the presented study. As structure-activity studies (performed by us) searching for plausible impact of BPs on observed toxicity failed, it even more strongly justifies the necessity to perform bioassays in order to study the real impact of such toxins and their mixtures on living organisms.

## 2. Results

The results of studies on toxicity and endocrine potential (and to subsequently select C_1_, C_2_ and C_3_ at, respectively, 33, 66 and 100% of EC_50_ of the given analytes) and determined impact of their co-presence on toxicity levels are listed in Table 1. Data on LOEC (lowest observable effect concentration) and NOEC (no-observed effect concentration) of a few given chemicals with respect to XenoScreen YES/YAS are also provided.

### 2.1. Structure-Toxicity Relationship

The results of bisphenol toxicity presented in Table 1 indicated that only 7 of 10 compounds expressed the detectable toxicity in the Microtox^®^ test, whereas BPM, BPP and BPZ were inactive. Therefore, this question was asked: What elements of the structure are responsible for the toxicity of these environmental pollutants? The results of molecular modeling performed with molecular dynamics are presented in Supplementary Table S2.

### 2.2. CA Studies

Results of CA studies as a function of MDR parameter values are summarized in Table 2a below.

### 2.3. IA Studies

Results of IA studies as a function of MDR parameter values are summarized in Table 2b below.

### 2.4. Regression Studies

Supplementary Table S3 presents the results of the comparison of the best-fit polynomial models (*y* = a*x*^2^ + b*x* + c) for toxicity studies of each couple of bisphenol analogues in the mode “effect of A on B” and “effect of B on A”.

### 2.5. MDR Uncertainties 

The MDR values presented enable us to provide uncertainties for the frequency distribution of the results provided (please refer to Table 3 for details) and to determine safety factors in cases of pollutants present in complex mixtures. In the case of substances with similar activity, the CA model had been shown to accurately predict the toxicity of the mixtures. Therefore, it can be considered as an advantage in environmental studies, as the most toxic/dangerous components of mixture can be easily targeted in this way.

## 3. Discussion

### 3.1. Discussion of Structure-Toxicity Relationship Results

We observed that the compounds active in the Microtox^®^ test expressed similar 3D structures (please refer to Supplementary Table S2 for details) for which:

(i) the distance between central carbon atom and oxygen atoms was close to 5.7 Å,
(ii) the distance between phenol oxygen atoms was equal to 9.3 Å,
(iii) the angle between phenol rings was near 109°.

On the other hand, bisphenols BPM and BPP with the additional aromatic ring between phenol fragments kept different shape of the molecules. They were nontoxic in the Microtox^®^ test; only slightly toxic BPZ out-stood the presented rule. Accordingly, 7 of 10 compounds were qualified to the following studies on the co-presence of analyte pairs in the Microtox test.

Results on the endocrine action obtained in XenoScreen YES/YAS endpoints/tests (Table 1) were difficult for the description with structure-activity relationships. Four compounds of different structures did not express activity in any of the performed tests. There were BPF, BPP, BPS and BPZ. Thus, only six compounds were active in one of the applied endocrine tests. BPA and BPC were the antagonist of estrogen receptor (YES+), whereas BPFL turned out to be the agonist of this receptor (YES−). On the other hand, two compounds, BPG and BPM expressed the agonistic action towards androgen receptor (YAS−). Compound BPE was extremely active in endocrine tests (the lowest value of LOEC), being a highly active agonist of estrogen as well as of androgen receptor.

### 3.2. Discussion on CA Studies

Studies with CA prove underestimation and synergism in most of the studied cases (only seven cases of overestimation were found, refer to Table 2a,b. for details). BPA combined with BPC, BPF, BFG and BPFL shows underestimation; however, interestingly, synergism with BPS is strongly present. Similar situations could be observed in the case of BPC’s impact when co-present with BPE, BPF and, in most cases, other analogues. Interestingly, again, single synergism is detected with BPG and BPFL, but most importantly in combination with BPS. BPE impact is synergistic with BPG and, again, with BPS at the lowest concentrations studied.

Binary mixtures of BPF and BPG show that there is a tendency to overestimate the behavior of these chemicals (with strong trend of increasing mutual impact with increase of concentrations of given compounds). BPF in the co-presence of BPS and BPFL shows very strong synergistic potential, even at the lowest concentration; also noticeable is a clear trend of concentrations impact on toxicity levels. Additionally, BPG in combination with BPS and BPFL in most cases shows concentration-dependent trend of signal underestimation (and synergism in the lowest concentration of BPG). A similar situation is observed for BPS-BPFL mixtures, where the impact of concentration is even more pronounced.

### 3.3. Discussion on IA Studies

As already stated, the results of MDR for IA modeling calculations reach generally higher values than CA models for similar mixtures [30,31]; this finding is confirmed in this study (refer to Table 2a,b for details). When analyzing the toxicity of analogues, IA models seem to deny the methodological background of the approach; even so, the results are presented to confirm the hypothesis stated. Model studies on BPA impact on other analogues’ toxicity quite accurately reflect the observed toxicity results. Despite studies with BPS, no clear impact of modulation of BPA concentrations on MDR is noticeable. The same holds true for the impact of BPC and BPE on other analogues studied.

Discrepancies are again noticeable in the studies on BPF impact on BPG and BPS solutions where antagonism and synergism, respectively, were noted with strong dependence on varying concentrations of analytes in binary mixtures. As already stated in CA studies, a mixture of BPF and BPG has a strong tendency for antagonistic behavior (certainly in studies with *Vibrio fischeri*), and a decrease of MDR is observable only for the highest concentration level of binary mixture ingredients in both CA and IA studies. The IA model seems to correctly predict the impact of BPF-BPFL mixture, as its MDR values oscillate around a value of “1.0”. Similar conclusions can be drawn in studies of BPG impact on BPS and BPFL and in the latter ones (BPS and BPFL) when present in a binary mixture.

### 3.4. Discussion of Regression Studies

Based on the results presented (ref. to Supplementary Table S3) it could be concluded that 18 out of 21 interactions displayed effects of A on B and B on A in similar manner; an independent mode of action is thus determined. In the remaining 3 out of 21 cases, the mode of action is different from the independent one. In general, these results coincide with the very significant number of cases with independent action found with application of the MDR approach.

The values and differences in the regression coefficients a, b and c and *R*^2^ for each one of the bisphenol analogue binary mixtures are presented in Supplementary Table S3. It is readily seen that the similarity between regression coefficients and *R*^2^ values within a binary mixture could be accepted as the “screening indication” for independent action mode. The differences in coefficient sign (change of slope) and differences higher than 0.1 in *R*^2^ are indications of behavior different from independent action.

Although the approach is semi-quantitative, it could be concluded that it might be of use for rapid estimation of the interactions, as follows:

Independent action: no differences in coefficients signs and model validity (*R*^2^ as measure) for the compared couples of bisphenols

Different from the independent action: differences in coefficients signs and model validity (differences in *R*^2^ higher than 0.1) for the compared couples of bisphenols

### 3.5. Environmental Impact

If one compares the toxicity ranges of the levels of different BPs studied in the present study (Table 1) with the concentration ranges found in environmental monitoring (surface an wastewater water in Asia [17,20,24], indoor dust in USA, China, Japan and Korea [32], it can be readily seen that the environmental levels are generally lower than those used in the present study. Certainly, the data presented refer to acute toxicity exposure, while environmental exposures are assumed to be of chronic character. The results of endocrine potential presented in this study for selected analytes reflect those reported by [24]. BPF and BPS are the most commonly used substitutes of BPA and, as confirmed in the present study, constitute similar threats to ecosystems, especially when their presence in complex mixtures with other pollutants is considered. Although the determining mode of action of the analytes of interest was not the aim of this research, one may conclude (in relation to other toxicity studies) that a competitive receptor-mediated mode of action for bisphenol analogues is very plausible. Although XenoScreen YES/YAS is a very potent tool for endocrine potential determination, one must consider its limitations resulting from the complexity of the procedure, which is of particular importance when ultra-low concentration levels of toxicants are studied. In such cases—To reflect the environmental threat of chronic exposure to low levels of stressors—it seems to be reasonable to introduce the study of toxicity of mixtures of pollutants (with a properly selected battery of bioassays) to routinely conducted environmental monitoring. In this study, we confirmed the impact of bisphenol A and its analogues on endocrine receptors—Even at 1.83 µM concentration levels. As expected, mostly estrogenic agonistic and androgenic antagonistic behaviors were observed. The levels studied reflect the environmentally stated levels of most BPs and constitute important prerequisite to run complex studies on the endocrine impact of these compounds, when present in binary (or higher) mixtures.

## 4. Materials and Methods 

The experimental design and approach was previously described in greater detail in [29], while the basics of the research performed are described below to assure easy access to a wider audience.

### 4.1. Chemicals and Reagents

Model substances selected for the study, BPA (CAS no. 80-05-7), BPC (CAS no. 79-97-0), BPE (CAS no. 2081-08-5), BPF (CAS no. 620-92-8), BPG (CAS no. 127-54-8), BPM (CAS no. 13595-25-0), BPP (CAS no. 2167-51-3), BPS (CAS no. 80-09-1), BPZ (CAS no. 843-55-0), and BPFL (CAS no. 3236-71-3) of analytical (>99%) purity were purchased from Sigma Aldrich (Darmstadt, Germany), as were HPLC grade methanol (CAS no. 67-56-1) and dimethyl sulfoxide (DMSO, CAS no. 67-68-5). Ultra-pure water was obtained using a grade A10 Milli-Q system (Millipore, Darmstadt, Germany) equipped with EDS-PAK^®^ Polisher cartridge (Merck, Darmstadt, Germany) to remove trace levels of bisphenol A and other endocrine-disrupting chemicals from water.

### 4.2. Standards and Mixtures Preparation

A standard stock solution of each compound was prepared separately by dissolving the given standard (to reach the concentration of 4 mg/mL) in HPLC grade methanol and stored in −20 °C. Various working solutions were obtained by serial dilution of the stock solutions with HPLC-grade methanol or ultrapure Milli-Q water (maximum methanol content in standard solutions for biological assays was 5%). The concentration ranges [µM] for bisphenol A analogues studied to determine their respective EC_50_ data and subsequently to select C_1_, C_2_ and C_3_ (being 33, 66 and 100% of EC_50_ of respective analyte) and to determine the impact of their co-presence on toxicity levels are listed in Table 1 (together with LOEC (lowest observable effect concentration) and NOEC (no-observed effect concentration) of given chemicals with respect to XenoScreen YES/YAS).

### 4.3. Microtox^®^ Reagents and Methodology

The Microtox^®^ test acute reagent (lyophilized *Vibrio fischeri*), osmotic adjustment solution (OAS, 22% solution of sodium chloride), reconstitution solution (RS), and diluent (2% solution of sodium chloride) were purchased from Modern Water (Cambridge, UK). The study was conducted using Microtox^®^ analyzer model 500 (M500, Modern Water, Cambridge, UK). The apparatus was equipped with 30 incubation wells as well as reagent (bacterial suspensions) and read wells. Temperatures were assigned to the corresponding type of performed test (in this case acute toxicity test) and internally maintained at 5.5 ± 1.0 °C for reagent well and 15.0 ± 0.5 °C for both the incubator part and the read well. pH was adjusted to fall within the 6.5–7.5 range with concentrated NaOH (CAS no. 1310-73-2) and HCl (CAS no. 7647-01-0) (purchased from Avantor Performance Materials S.A. (Poznań, Poland)) using Metrohm pH-meter model 827 (Metrohm, Opacz-Kolonia, Poland).

The EC_50_ parameter for each analyte of interest separately was determined by standard protocol using the Microtox^®^ Analyzer Model 500 and serial dilutions. Lyophilized reagent with *Vibrio fischeri* bacteria was hydrated with 1 mL of RS and maintained at 5.5 ± 1.0 °C, subsequently 100 µL of bacterial solution and a pre-made samples of standard dissolved in distilled water (made from stock solutions of given analyte dissolved in ethanol) were added into the vials. To produce a suitable osmotic pressure (above 2%), OAS was added to the vial with the highest concentration and proper dilutions and ions additions were prepared. The incubation time was 30 min. Range-screening test for insoluble substance was also performed to narrow the range of concentrations tested; afterwards, proper tests were performed in triplicates to determine the range of linearity and calculate particular analytes EC_50_ values.

In order to determine whether the addition of one BP to solution of another one would change the bioluminescence of bacterial suspension, concentrated solutions of the compounds were prepared. Test mixtures were prepared in such a way that the compounds were present in an appropriate ratio: 100% of the first model substance and the second substance with a reduced effect to 33% and 66% of EC_50_. Incubation time of samples with bacteria for all of the tests was 30 min.

### 4.4. XenoScreen YES/YAS Reagents and Methodology

A set of XenoScreen YES/YAS reagents was purchased from Xenometrix AG (Allschwil, Switzerland), namely vial with hERα yeasts (to determine estrogenic activity) and hAR (to determine androgenic activity) settled on the filtration paper, basal medium, vitamin solution, l-aspartic acid solution, l-treonine solution, CuSO_4_, 17β-estradiol (E2, YES+ control), 5α-dihydrotestosterone (DHT, YAS+ control), 4-hydroksytamoxyphene (HT, YES− control), flutamide (FL, YAS− control), DMSO. CPRG (chlorophenol red-β-d-galactopyranoside) was purchased from Sigma Aldrich (Hamburg, Germany). Measurement of cell density (wavelength 690 nm) and the intensity of the CPRG transformation product (wavelength 570 nm) was performed with a TECAN Infinite M200 spectrophotometer (Tecan Group Ltd., Männedorf, Switzerland).

To investigate endocrine potential of bisphenol analogues, a slightly modified protocol of XenoScreen YES/YAS was utilized, which uses genetically modified yeast cells of *Saccharomyces cerevisiae*. For this purpose, the DNA sequence of human estrogen hERα or androgen hAR receptors was stably integrated into the main chromosome of the yeast cells. Yeasts exposed to compounds that act endocranially produce β-galactosidase, which oxidizes the dye CPRG in growth medium. The interpretation occurs by measuring the density of the cell suspension and the color saturation of the oxidized dye. Furthermore, the cells also contain an expression plasmid carrying the lacZ reporter gene encoding the enzyme β-galactosidase and means responsive to estrogens (YES) or androgen (YAS). The yeast cells were cultured from the filter papers in growth medium (basic medium with a vitamin solution, solution of l-threonine, l-aspartic acid and copper (II) sulfate (VI)). 5 mL of growth medium was transferred to a labeled culture bottles with caps with a gas permeable filter; afterwards, the yeast disks were sterilely transferred and placed on an orbital shaker set at 32 °C and 100 rpm for 48 h. 100 µL of DMSO was added to each control vial containing standards: E2 (17β-estradiol control of YES agonist), DHT (5α-dihydrotestosterone control of YAS agonist), HT (4-hydroxytamoxifen control of YES antagonist), and FL (flutamide control of YAS antagonist). Test plates were prepared in such a way that the controls were in duplicate in eight serial dilutions, respectively:

- YES Agonist plate E2 (min. concentration 1 × 10^−11^ M, max. concentration 1 × 10^−8^ M).
- YES Antagonist plate HT (min. concentration 1 × 10^−8^ M, max. concentration 1 × 10^−5^ M, additionally in the entire plate E2 was present at constant concentration of 1×10^−9^ M).
- YAS Agonist plate DHT (min. concentration 1 × 10^−9^ M, max. concentration 1 × 10^−6^ M).
- YAS Antagonist plate FL (min. concentration 1 × 10^−7^ M, max. concentration 1 × 10^−4^ M, additionally in the entire plate DHT was present at constant concentration of 3 × 10^−8^ M).

The addition of E2 or DHT present at the same concentration to the entire YES or YAS antagonist plate, respectively, is intended to examine (confirm/deny) andro- and estrogenic antagonistic activity of samples. A substance with the antagonist properties competes with E2 or DHT present on the plate and binds to the receptor without inducing the expression of β-galactosidase. Without the enzyme, substrate staining does not occur; however, if the test sample does not contain antagonistic substances, then E2 and DHT present in the wells bind with the receptor expressing β-galactosidase and staining of the substrate occurs.

60 µL of 6 mM CRPG dye was added to each assay well. BPs′ serial dilutions were studied to detect a broad range of possible interactions. All of the studies on mixtures were performed in triplicates; furthermore, controls were made for pure substances in duplicates. 100 µL of YES and YAS suspension of yeast culture (yeast cells density > 0.3 OD_690_) was added into agonist and antagonist YES and YAS plates, respectively. Assay plates were sealed with semi-permeable membranes and placed in a zipper bag moistened with watered gauze on an orbital shaker for 48 h at 32 °C 100 rpm. After 48 h of incubation, a cell density (by OD) was read at a wavelength of 690 nm, and color intensity at a wavelength of 570 nm was determined. Afterwards, the activity of β-galactosidase was calculated as ratio of [(OD_570_ − OD_690_)/OD_690_].

### 4.5. Calculations of Model Deviation Ratios (MDRs)

The two most exploited models for environmental hazard and risk assessments of mixtures are Concentration Addition (CA) and Independent Action (IA) [29]. These two approaches could assess the combined toxicological effect of chemicals assuming similar mode of action (CA) or dissimilar mode of action (IA). In the environmental risk assessment, CA models are more frequently applied, since they are slightly more conservative than IA models and could be used as a precautious first tier for environmental hazard and risk assessment of mixtures, irrespective of the modes of action of their components.

In this study, the combined toxicological effect of mixture was assessed by a CA model using Equation (1) [27]:

(1) $$ECx_{mix}={\left(\overset{n}{\underset{i=1}{\sum }}\frac{p_{i}}{ECx_{i}}\right)}^{-1}$$

where *ECx_mix_* is the total concentration of the mixture that causes *x* effect; *p_i_* indicates the proportion of component *i* in the mixture; *n* indicates the number of components in the mixture; *ECx_i_* indicates the concentration of component *i* that would cause *x* effect.

The independent action (IA) model is used to test toxicants in a mixture for a dissimilar mode of action. The concept is that they act independently. In fact, the IA model is a statistical approach to predict the chance that one of multiple events will occur. The total mixture effect is calculated using Equation (2):

(2) $$\left(c_{mix}\right)=1-\overset{n}{\underset{i=1}{\prod }}\left(1-E\left(c_{i}\right)\right)$$

where *E(c_mix_)* is the total concentration of the mixture; *E(c_i_)* is the concentration expected from component *i*.

The CA model does not count for possible interaction between different chemicals in the mixture and deviations of tested mixture toxicity from the predicted one could be evidence for synergistic or antagonistic interaction between chemicals. To outline significant deviations (interactions between chemicals), the model deviation ratio (MDR) approach proposed by [31] is applied. MDR (unitless) is defined as Equation (3):

(3) $$\mathrm{MDR}=\frac{Expected\;toxicity}{observed\;toxicity}$$

where *Expected toxicity* is the effective concentration toxicity for the mixture predicted by CA/IA model; *Observed toxicity* is the effective concentration toxicity for the mixture obtained from toxicity testing.

The MDR values are easily applicable to reflect the impact of toxicants mixture, when compared to predictive models. MDRs can be also presented in a plot form on a logarithmic scale to visualize the predicted toxicity in comparison to an observed one. The mixtures with MDR values falling outside the range from 0.5 to 2.0 have a high probability for biologically significant, respectively, synergistic or antagonistic interactions between chemicals. The underestimated or overestimated toxicity mixtures close to these levels also most likely include possible synergistic or antagonistic interactions [31]. In current research, it was arbitrarily assumed that MDR falling within 0.50–0.71 and 1.40–2.00 justify the concluding on, respectively, possible under- and overestimation of presented models.

Since CA and IA models are one of many options for assessing possible interactions between the chemicals involved in the ecotoxicity study, we have tried another approach for estimation of the possible independent action and action different from independent. This simple mode of assessment requires calculation of the regression function A = f(B), where A and B are notations for two different bisphenol analogues as well as regression B = f(A). If in the concentration intervals of A and B experimentally studied, the slope and the offset of the polynomial equation do not differ significantly, one can accept that there is independent action mode at hand. Different mode of action (dependent mode) is ascribed if the regression parameters are significantly different. A similar approach (best-fit modeling) proved to be effective in the assessment of the bisphenol analogues’ interactions [29].

### 4.6. Molecular Modeling Calculations

To determine the most probable conformations of the studied bisphenols and its analogues, two molecular modeling techniques were used. Each compound first underwent molecular dynamics calculation for at least 200 ps after thermal equilibration at 300 K and the most popular conformer was selected. The geometry of this conformer was next optimized by molecular mechanics to minimize its potential energy. The Polak-Ribiere algorithm with termination at 0.05 kcal/(Å·mol) RMS (root mean squared) gradient was used in molecular mechanics’ optimization. The modeling calculations were done with HyperChem 8.0 software (Hypercube, Inc., Gainesville, FL, USA) using Bio+(CHARMM) empirical force field.

### 4.7. Quality Assurance/Quality Control

For quality assurance of running the proper test, the following parameters were used, according to the manufacturers’ guidelines: for Microtox^®^, I_0_ of bacterial suspension >70 U (chromium sulfate was used as a positive control in the bacterial stock suspension test run), and for XenoScreen YES/YAS, the OD_690_ of yeast cultures should be >0.3. In all cases presented, these criteria were fulfilled.

## 5. Conclusions

The correct evaluation of the interactions between potentially toxic (ecotoxic) materials is a significant challenge to all professionals dealing with hazardous materials. In the present work, two simple options for rapid assessment of the ecotoxicity of BPs in their binary mixtures were studied. According to the MDR approach, most of the binary mixtures revealed predominantly independent modes of action; however, several cases showed a typical synergistic or antagonistic effect. We confirmed that it is possible to introduce a relative scale for the calculated MDR values to better distinguish (even qualitatively rather than quantitatively) the independent, synergistic or antagonistic effects.

The calculation of the best-fit polynomial regression models for the impact of A member of the mixture on the B member (and vice versa) makes it possible to compare/distinguish independent interactions from dependent ones. In principle, the calculation of the linear regression models approach confirmed the dominant number of independent action in binary mixtures of bisphenol analogues (18 out of 21 mixtures).

Since studies on mixture toxicity of newly synthesized chemicals remain scarce, the data presented constitute an important record for environmental toxicologists. It should be highlighted that these analytes are of the highest probability of synergistic or antagonistic interactions. Moreover, there is a risk that BPs can be present in some environmental compartments at higher concentrations that have not been examined so far. The results presented here offer clear guidance on how to predict the combined effect of BPs (to endocrine systems and bacteria) in their binary mixtures. Further investigations are required to better understand and mathematically describe the behaviors of pollutants present in environmentally relevant mixtures, which lead to various acute/chronic endpoints for communities of geographically variable characteristics for given regions.

## Figures and Tables

**Table 1:** Concentration levels of bisphenol analogues studied during the research and EC_50_ values calculated for respective compounds.

Analyte	Microtox^®^ *					XenoScreen YES/YAS				
	Concentration Ranges Tested	EC_50_ ± SD (*n* = 3)	C_1_	C_2_	C_3_	Concentration Ranges Tested	Effect (*n* = 3)			
	[µM]					[µM]				
							YES+	YES−	YAS+	YAS−
BPA	8.96–71.68	37.7 ± 6.7	12.44	24.88	37.69	1.71–1752.15	173.9 ^L^	>1752 ^N^	>1752 ^N^	>1752 ^N^
BPC	6.33–63.84	30.8 ± 2.1	10.17	20.34	30.82	1.53–1560.43	15.4 ^L^	>1560 ^N^	>1560 ^N^	>1560 ^N^
BPE	38.19–305.49	57.9 ± 4.4	19.12	38.24	57.94	1.83–1866.89	>1886 ^N^	1.83 ^L^	>1886 ^N^	1.83 ^L^
BPF	10.22–81.72	28.3 ± 1.5	9.34	18.68	28.31	1.95–1997.70	>1997 ^N^	>1997 ^N^	>1997 ^N^	>1997 ^N^
BPG	2.60–26.19	14.85 ± 0.98	4.90	9.80	14.85	1.25–1280.21	>1280 ^N^	>1280 ^N^	>1280 ^N^	12.6 ^L^
BPM	70.84–566.77	>566.77 ^#^	-	-	-	1.13–1154.53	>1154 ^N^	>1154 ^N^	>1154 ^N^	11.5 ^L^
BPP	70.84–566.77	>566.77 ^#^	-	-	-	1.13–1154.53	>1154 ^N^	>1154 ^N^	>1154 ^N^	>1154 ^N^
BPS	32.69–261.54	61.6 ± 5.6	20.34	40.68	61.64	1.56–1598.27	>1598 ^N^	>1598 ^N^	>1598 ^N^	>1598 ^N^
BPZ	75.62–762.23	>762.23 ^#^	-	-	-	1.46–1490.60	>1490 ^N^	>1490 ^N^	>1490 ^N^	>1490 ^N^
BPFL	0.58–58.40	3.31 ± 0.52	1.09	2.18	3.31	1.12–1141.52	1141.5 ^L^	113.3 ^L^	1141.5 ^L^	>1141 ^N^

* C_1_, C_2_ and C_3_ stand for 33, 66 and 100% of EC_50_ of respective analyte. ^#^ solubility limit reached under conditions of the experiment, ^L^ LOEC, ^N^ NOEC.

**Table 2a:** MDR values for bisphenol A analogues binary mixtures toxicity studies performed with Microtox^®^ assay (for both CA modelling) (red—Synergism, blue—Antagonism, green—Overestimation, yellow—Underestimation, C_1_, C_2_ and C_3_ stand for 33, 66 and 100% of EC_50_ of respective analogue as presented in Table 1. #—data inconclusive).

		**Concentration Addition**																	
	
		BPA																	
		C_1_	C_2_	C_3_															
C_1_	BPC	0.76	0.71	0.74															
C_2_		0.79	0.79	0.75	BPC														
C_3_		0.90	0.81	0.59	C_1_	C_2_	C_3_												
C_1_	BPE	0.28	0.30	0.30	0.60	0.64	0.63												
C_2_		0.74	0.65	0.77	0.84	0.71	0.58	BPE											
C_3_		0.78	0.71	0.46	0.78	0.81	0.56	C_1_	C_2_	C_3_									
C_1_	BPF	0.80	0.76	0.79	0.74	0.77	0.81	0.68	0.73	0.86									
C_2_		0.82	0.81	0.82	0.76	0.90	0.79	0.70	0.72	0.83	BPF								
C_3_		0.87	0.82	0.64	0.75	0.80	0.63	0.69	0.73	0.63	C_1_	C_2_	C_3_						
C_1_	BPG	0.64	0.66	0.70	0.49	0.61	0.50	0.42	0.47	0.53	#	1.73	1.24						
C_2_		0.67	0.68	0.69	0.52	0.72	0.57	0.53	0.63	0.69	0.67	1.32	0.55	BPG					
C_3_		0.74	0.71	0.69	0.56	0.68	0.53	0.52	0.61	0.61	1.34	0.75	0.37	C_1_	C_2_	C_3_			
C_1_	BPS	0.54	0.67	0.71	0.46	0.45	0.42	0.48	0.46	0.51	0.11	0.22	0.41	0.70	0.60	0.55			
C_2_		0.36	0.44	0.40	0.75	0.67	0.64	0.52	0.63	0.67	0.09	0.12	0.39	0.62	0.53	0.58	BPS		
C_3_		0.55	0.58	0.35	0.73	0.71	0.47	0.75	0.74	0.56	0.08	0.12	0.17	0.99	0.76	0.58	C_1_	C_2_	C_3_
C_1_	BPFL	1.03	1.33	0.9	0.73	0.64	0.71	1.00	0.87	0.88	0.21	0.42	0.84	0.93	0.65	0.70	0.35	0.85	0.68
C_2_		0.77	0.79	0.79	0.54	0.6	0.68	0.72	0.77	0.83	0.04	0.17	0.31	0.48	0.53	0.59	0.29	0.48	0.74
C_3_		0.66	0.64	0.61	0.37	0.52	0.65	0.52	0.6	0.59	0.02	0.09	0.16	0.44	0.51	0.57	0.21	0.35	0.44

**Table 2b:** MDR values for bisphenol A analogues binary mixtures toxicity studies performed with Microtox^®^ assay (for IA modelling) (red—Synergism, blue—Antagonism, green—Overestimation, yellow—Underestimation, C_1_, C_2_ and C_3_ stand for 33, 66 and 100% of EC_50_ of respective analogue as presented in Table 1. #—data inconclusive).

		**Independent Action**																	
	
		BPA																	
		C_1_	C_2_	C_3_															
C_1_	BPC	1.31	1.23	1.29															
C_2_		1.11	1.19	1.17	BPC														
C_3_		1.09	1.09	0.83	C_1_	C_2_	C_3_												
C_1_	BPE	1.58	1.44	1.32	1.07	1.27	1.10												
C_2_		1.41	1.22	1.20	1.18	1.10	1.16	BPE											
C_3_		1.26	1.40	0.76	1.14	0.89	0.80	C_1_	C_2_	C_3_									
C_1_	BPF	1.22	1.18	1.19	1.24	1.20	1.10	1.14	1.16	1.10									
C_2_		1.10	1.16	1.14	1.15	1.31	1.13	1.09	1.11	1.10	BPF								
C_3_		1.09	1.14	0.88	1.14	1.11	0.87	1.15	1.13	0.86	C_1_	C_2_	C_3_						
C_1_	BPG	1.13	1.01	1.04	0.95	0.91	0.95	0.87	0.95	0.96	#	1.61	3.40						
C_2_		1.00	0.96	0.97	1.28	1.06	0.98	0.90	0.95	0.95	2.50	2.80	1.72	BPG					
C_3_		0.98	0.92	0.91	0.87	0.83	0.77	0.94	0.94	0.86	1.64	1.17	0.87	C_1_	C_2_	C_3_			
C_1_	BPS	0.95	0.72	0.89	0.90	1.11	1.08	1.05	0.87	1.11	0.63	0.49	0.48	1.17	1.01	1.31			
C_2_		0.72	1.14	1.07	0.98	1.10	1.09	0.94	1.05	1.10	0.67	0.37	0.40	1.09	0.98	1.09	BPS		
C_3_		1.24	1.08	0.68	0.96	1.08	0.73	1.04	1.10	0.81	0.94	0.85	0.32	1.05	1.16	0.88	C_1_	C_2_	C_3_
C_1_	BPFL	1.38	1.10	0.96	1.08	0.99	0.85	1.36	1.20	1.02	0.98	0.96	1.13	1.48	0.80	0.76	0.78	0.91	0.83
C_2_		1.67	1.04	0.90	0.85	0.86	0.85	1.06	1.07	0.95	0.78	1.06	1.09	0.90	0.77	0.76	1.28	0.90	0.78
C_3_		1.08	0.98	0.80	0.89	0.86	0.88	1.03	1.07	0.85	1.19	0.99	0.87	0.91	0.80	0.79	0.85	1.05	0.70

**Table 3:** Percentile values for model deviation ratios (MDR) and numbers of cases for each group of CA and IA experiments of BP toxicity studies.

Model	No. of Cases							
	Synergism	Under-estimation	Over-estimation	Antagonism	Percentile			
					80	90	95	99
CA	45	78	1	0	0.464	0.338	0.170	0.075
IA	5	4	8	3	0.860	0.780	0.720	0.396

## Supplementary Materials

The following are available online, Supplementary Table S1: Basic information about analytes studied, Supplementary Table S2: Structural parameters of bisphenol analogs calculated with molecular dynamics, Supplementary Table S3: Comparison of the polynomial models for BPs studied with Microtox^®^.
