# Supplementary material for: Binary Mixtures of Selected Bisphenols in the Environment: Their Toxicity in Relationship to Individual Constituents
Source: Molecules. 2018 Dec 6;23(12):3226. doi: 10.3390/molecules23123226 (PMC6321295; doi:10.3390/molecules23123226)
Supplement: Supplementary file 1 [file molecules-23-03226-s001.pdf]

Electronic supplementary material for Article

Binary mixtures of selected bisphenols in the environment: their toxicity in relationship to individual constituents

Katarzyna Owczarek[1], Błażej Kudłak[1,*], Vasil Simeonov[2], Zofia Mazerska[3], Jacek Namieśnik[1]

[1] Department of Analytical Chemistry, Faculty of Chemistry, Gdansk University of Technology, 11/12 Narutowicza Str., Gdańsk 80-233, Poland; blakudla@pg.edu.pl

[2] Department of Analytical Chemistry, Faculty of Chemistry and Pharmacy, University of Sofia „St. Kliment Ohridski", 1164 Sofia, 1 James Bourchier Blvd., Bulgaria; vsimeonov@chem.uni-sofia.bg

[3] Department of Pharmaceutical Technology and Biochemistry, Faculty of Chemistry, Gdansk University of Technology, 11/12 Narutowicza Str., Gdańsk 80-233, Poland; zofia.mazerska@pg.edu.pl

***** Correspondence: blakudla@pg.edu.pl; phone no.: +48-58-347-2128, ORCID no. 0000-0002-2237-2927

Supplementary Table S1. Basic information about analytes studied

Analyte /CAS no./ molecular weight [g/mol]	Structure	IUPAC name	Application (if known)
**BPA** / 80-05-7/ 228.29	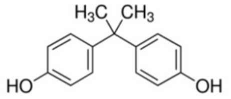	2,2-bis(4-hydroxyphenyl) propane	Food packaging coatings, plastic bottles, plastic items (toys, every-day use products) [I]
**BPC** /79-97-0/ 256.34	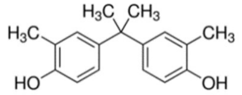	2,2-bis(4-hydroxy-3-methylphenyl) propane	Used as intermediate in pesticide and pharmaceuticals production [II]
**BPE** /2081-08-5/ 214.26	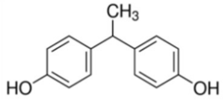	1,1-bis(4-hydroxyphenyl) ethane	Polymer production, flame retardants resin composite, heat sensitive recording material [III, IV]
**BPF** /620-92-8/ 200.23	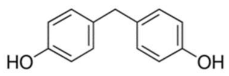	4,4′-methylenediphenol	BPA substitute, plastic items (toys, every-day use products)
**BPG** /127-54-8/ 312.45	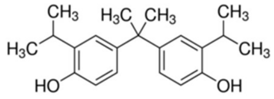	2,2-bis(4-hydroxy-3-isopropylphenyl) propane	Thermally responsive recording materials [V]
**BPS** /80-09-1/ 250.27	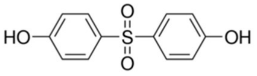	4,4′-sulfonyldiphenol	Dental sealants, thermal papers production, electronics [VI]
**BPFL** /3236-71-3/ 350.41	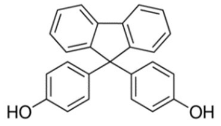	4,4′-(9-fluorenylidene)diphenol	Photosensitive resins [VII]

**BPZ**<br><br>/843-55-0/ 268.35	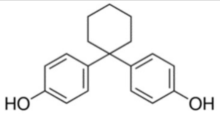	4,4′-cyclohexylidene-bisphenol	Polycarbonates plastic production, thermosensitive materials, optical composites [VIII, IX]
**BPM**<br><br>/13595-25-0/ 346.46	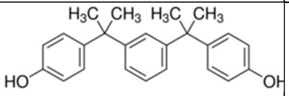	4,4′-(1,3-phenylenediisopro-pylidene)bisphenol	Flame retardants, polycarbonate plastics production, aromatic co-polyesters manufacturing, thermosensitive materials [X, XI]
**BPP**<br><br>/2167-51-3/ 346.46	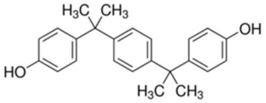	4,4′-(1,4-phenylenediisopropylidene) bisphenol	Found in food packaging, polycarbonate plastics production, flame retardant production, thermoplastic resins production [XII, XIII, XIV]

Supplementary Table S2. Structural parameters of bisphenol analogs calculated with molecular dynamics

Analyte	Structure	Distance [Å]		Angle [°]
		O-C[1]	O-O[2]	O-C-O[3]
**BPA**	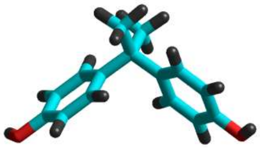	5.74	9.25	107.26
**BPC**	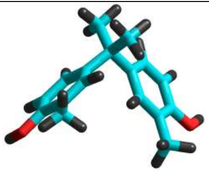	5.74	9.19	106.38
**BPE**	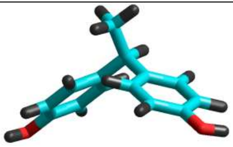	5.72	9.29	108.47
**BPF**	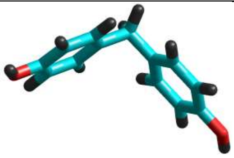	5.71	9.29	109.00
**BPG**	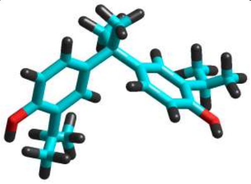	5.74	9.19	106.55
**BPS**	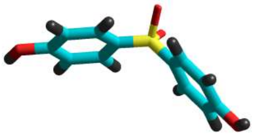	5.67	10.26	129.41
**BPFL**	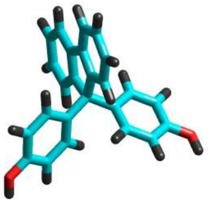	5.74	9.70	115.40
**BPZ**	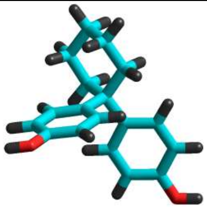	5.76	9.11	104.36

**BPM**	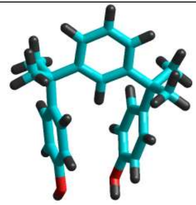	5.92	3.50	34.41
**BPP**	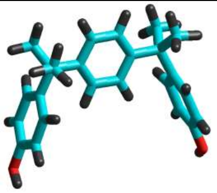	6.38	9.73	99.2

[1] Distance between central carbon atom and left branch oxygen atom forming phenol
[2] Distance between both branches oxygen atoms forming phenols.
[3] The angle between phenolic rings

Supplementary Table S3. Comparison of the polynomial models for BPs studied with Microtox®

BPs binary combination y/x	Regression coefficients: a, b, c	Correlation R^2	Mode of action
BPA/BPC	0.06; -0.16; 0.71	0.97	IA
BPC/BPA	0.07; -0.21; 0.72	0.98	
BPA/BPE	0.12; -0.44; 0.84	0.94	IA
BPE/BPA	0.08; -0.34; 0.52	0.97	
BPA/BPF	0.07; -0.27; 0.93	0.98	IA
BPF/BPA	0.06; -0.21; 0.85	0.98	
BPA/BPG	0.005; 0.08; 0.58	0.97	IA
BPG/BPA	0.007; 0.12; 0.61	1.00	
BPA/BPS	0.11; -0.42; 0.87	0.97	IA
BPS/BPA	0.11; -0.12; 0.36	0.96	
BPA/BPFL	0.06; -0.17; 075	0.98	IA
BPFL/BPA	0.04; -0.27; 0.16	0.98	
BPC/BPE	0.03; 0.002; 0.48	0.96	IA
BPE/BPC	0.07; 0.05; 0.60	0.94	
BPC/BPF	0.04; -0.06; 0.66	0.96	IA
BPF/BPC	0.05; -0.12; 0.73	0.97	
BPC/BPG	-0.003; 0.17;0.36	0.99	Different from IA
BPG/BPC	0.05; -0.16; 0.39	0.97	

BPC/BPS	0.06; -0.19; 0.66	0.94	IA
BPS/BPC	0.08; -0.24; 0.65	0.97	
BPC/BPFL	-0.007; 0.16; 0.49	0.99	IA
BPFL/BPC	-0.005; 0.26; 0.36	0.99	
BPE/BPF	0.05; -0.15; 0.75	0.97	IA
BPF/BPE	0.05; -0.14; 0.72	0.97	
BPE/BPG	0.02; 0.02; 0.55	0.99	IA
BPG/BPE	0.03; 0.11; 0.49	0.99	
BPE/BPS	0.07; -0.24; 0.79	0.96	IA
BPS/BPG	0.07; -0.18; 0.65	0.96	
BPE/BPFL	0.07; -0.19;0.73	0.98	IA
BPFL/BPE	0.06; -0.11; 0.62	0.99	
BPF/BPG	0.04; -0.10; 0.72	0.99	IA
BPG/BPF	0.04; -0.15; 0.92	0.99	
BPF/BPS	0.15; -0.48;0.58	0.95	IA
BPS/BPF	0.11; -0.25; 0,41	0.96	
BPF/BPFL	0.12; -0.32; 0.34	0.99	IA
BPFL/BPF	0.08; -0.33; 0.92	0.99	
BPG/BPS	0.06; -0.22; 0.92	0.77	Different from IA
BPS/BPG	0.07; -0.20; 0.68	0.98	
BPG/BPFL	-0.02; 0.22; 0.38	0.99	IA
BPFL/BPG	-0.01; 0.59; -0.005	0.99	
BPS/BPFL	0.05; -0.11; 0.53	0.86	Different from IA
BPFL/BPS	0.01; 0.10; 0.34	0.99	

References for electronic supplement:

[I] Vogel, S. The Politics of Plastics: The Making and Unmaking of Bisphenol A "Safety". *Am. J. Publ. Health* **2009**, *99*, 559–566.

[II] Rudd, J.F. Blends of Phenolphthalein Polycarbonates with Rubber-Modified Monovinylidene Aromatic Copolymers, Eur. Pat. Appl. 0000146, **1979**.

[III] Hada, T.; Shimizu, M.; Aoki, Y.; Endo, N.; Ohhashi, H.; Ishiguro, T., Heat-Sensitive Recording Material and Method for Producing Same. U.S. Patent, 13,254,045, 16 February **2010**.

[IV] Zouta, K.; Tokita, A. Polycarbonate Resin Composition, and Fluorescence Detection/analysis Substrate Produced using Polycarbonate Resin Composition. U.S. Patent, 14,901,210, 19 June **2014**.

[V] Niederst, J.; Evans, R.H.; O'Brien, R.M.; Romagnoli, K.; Killilea, H.T.; Von Maier, M.S.; Deng, L. Developer for Thermally Responsive Record Materials. U.S. Patent 14,418,014, 15 March **2013**.

[VI] Chen, D.; Kannan, K.; Tan, H.; Zheng, Z.; Feng, Y.L.; Wu, Y.; Widelka, M. Bisphenol analogues other than BPA: Environmental occurrence, human exposure and toxicity—A review. *Environ. Sci. Technol.* **2016**, *50*, 5438–5453.
[VII] Hsieh, B.Y.; Hsu, J.P. Photosensitive Resin Composition, Color Filter and Method for Manufacturing the Same, and Liquid Crystal Display Apparatus. U.S. Patent 14,554,067, 26 November **2014**.
[VIII] Ochiai, T.; Masuda, T. Thermal Recording Material and Method for Producing the Same. U.S. Patent 13,132,984, 21 November **2009**.
[IX] Coleman, Z. **2011**. Method of manufacturing an optical composite, U.S. Patent 13/163,938.
[X] Zouta, K.; Nakao, K.; Takagi, Y.; Ishii, H. Flame-Retardant Sheet or Film, Products Comprising the Same and Process for Production Thereof, U.S. Patent 14,900,386, 20 June **2011.**
[XI] Weiss, J. Process for Improving the Performance of a Marine Antifouling Paint, and Top Coat Composition Employed in this Process. Eur. Pat. Appl. 0005771, **1979**.
[XII] Wagner, S.; Kramer, R.H.; Konig, A.; Roth, M. Flame-Retardant Polyesters. U.S. Patent 14,917,039, 3 December **2015**.
[XIII] Tamura, M.; Ohashi, T.; Enomoto, T. Thermosetting Resin Composition Containing Polymer Having Specific Terminal Structure. U.S. Patent 14,900,489, 4 June **2014**.
[XIV] Teichert, D.; Conrad, L.; Grotzinger, J. Epoxy Resin-Upgraded Cement-Bound Composition as Coating or Seal. U.S. Patent 14,904,884, 14 July **2014**.
